# D Quantification of Tumor Vasculature in Lymphoma Xenografts in NOD/SCID Mice Allows to Detect Differences among Vascular-Targeted Therapies

**DOI:** 10.1371/journal.pone.0059691

**Published:** 2013-03-26

**Authors:** Marco Righi, Arianna Giacomini, Loredana Cleris, Carmelo Carlo-Stella

**Affiliations:** 1 Institute of Neuroscience, Consiglio Nazionale delle Ricerche, Milan, Italy; 2 Department of Medical Biotechnology and Translational Medicine, University of Milano, Milan, Italy; 3 Department of Oncology and Hematology, Humanitas Clinical and Research Center, Rozzano, Milan, Italy; 4 Experimental Oncology, Fondazione IRCCS Istituto Nazionale dei Tumori, Milan, Italy; Okayama University, Japan

## Abstract

Quantitative characterization of the *in vivo* effects of vascular-targeted therapies on tumor vessels is hampered by the absence of useful 3D vascular network descriptors aside from microvessel density. In this study, we extended the quantification of planar vessel distribution to the analysis of vascular volumes by studying the effects of antiangiogenic (sorafenib and sunitinib) or antivascular (combretastatin A4 phosphate) treatments on the quantity and spatial distributions of thin microvessels. These observations were restricted to perinecrotic areas of treated human multiple myeloma tumors xenografted in immunodeficient mice and to microvessels with an approximate cross-sectional area lower than 75 µm^2^. Finally, vessel skeletonization minimized artifacts due to possible differential wall staining and allowed a comparison of the various treatment effects. Antiangiogenic drug treatment reduced the number of vessels of every caliber (at least 2-fold fewer vessels vs. controls; p<0.001, n = 8) and caused a heterogeneous distribution of the remaining vessels. In contrast, the effects of combretastatin A4 phosphate mainly appeared to be restricted to a homogeneous reduction in the number of thin microvessels (not more than 2-fold less vs. controls; p<0.001, n = 8) with marginal effects on spatial distribution. Unexpectedly, these results also highlighted a strict relationship between microvessel quantity, distribution and cross-sectional area. Treatment-specific changes in the curves describing this relationship were consistent with the effects ascribed to the different drugs. This finding suggests that our results can highlight differences among vascular-targeted therapies, providing hints on the processes underlying sample vascularization together with the detailed characterization of a pathological vascular tree.

## Introduction

Beginning with the pioneering work of Folkman [1], increasing attention has been paid to the supportive role played by tumor vessels in providing proliferating tumor cells with nutriments and oxygen [2]. Although the complete destruction of the tumor vasculature cannot yet be achieved, efforts in this direction have contributed to the development of an increasing number of vascular-targeted therapies [3]. These therapies are based on either antiangiogenic drugs, which usually block the development of new vessels by inhibiting the VEGF pathway [4], or antivascular agents, which destabilize established vessels causing collapse [5]. Recently, with the discovery of the vasculature normalizing effect [6], [7], it has been shown that antiangiogenic drugs can promote improved delivery of drugs to tumoral tissues [8], [9]. Furthermore, these results have stimulated the search for combined therapies in which antiangiogenic and antivascular drugs or conventional chemotherapies synergize to efficiently destroy the tumor vasculature [10].

Unfortunately, quantification of the efficiency of these therapies is hampered by the inability to exhaustively describe the arrangement of a vascular network using a limited number of efficient parameters. In the last 2 years, efforts aimed at the unbiased quantitation of vascular beds have resulted in a number of computer-based utilities addressing planar images or 3D image stacks. Among these utilities, the CAIMAN [11], AngioTool [12] and RAVE [13] programs can be used to calculate a number of parameters related to the quantity and length of vessels. In addition, these applications can be used to quantitate wall thickness, branching index values, fractal dimensions and 3D branch angle. Using a different approach, Kocińsky and coworkers [14] proposed that 3D texture analysis is a way to address and quantify the physical parameters of actual vascular beds. However, these textural descriptors lack a geometric interpretation with respect to vascular tree structure. Therefore, despite these efforts, quantifying vascular characteristics that can be directly observed, *i.e.*, correlating visual observations with mathematical values, remains difficult. In this respect, only parameters that estimate vessel characteristics, such as mean vessel density (MVD) [15], [16], are widely used to compare the effects of different *in vivo* therapeutic approaches in whole tumor nodules.

However, we observed that antiangiogenic drugs affect not only the total vessel quantity but also their spatial arrangement [17], which leads to a heterogeneous distribution of vessels in the treated vascular networks. Building upon this observation, we focused on thin microvessels with an apparent diameter of less than 10 µm for 2 reasons: first, microvessels ensure the delivery of oxygen and nutrients throughout the tissue more so than larger vessels [18] and second, the even distribution of microvessels throughout the tumor suggests that their presence/absence could signal even small changes in tumor vascularization. Thus, we investigated whether tumor vascular trees treated with different vascular-targeted therapies could be differentiated based solely on the amount, distribution and caliber of thin microvessels. In this respect we calculated the 3D spatial dispersion of vessels by assessing the number of pixel-dilation cycles needed to fill-up a pre-defined portion (90%) of each vascular volume. This approach, previously applied to 2D images [17], allowed in this work to discriminate between homogeneous or clusterized 3D vessel distributions. Herein, we report that vascular amounts, distributions and calibers were linked by an evident, although unexpected, relationship. The shape of the curve describing this relationship appeared to change according to the treatment applied and provided a graphical and quantitative characterization of the vascular bed under analysis.

## Methods

### Ethics Statement

Animal experiments were performed according to Italian law (D.L. 116/92 and following additions), which enforce the EU 86/109 Directive, and were approved by the Ethical Committee for Animal Experimentation of the National Cancer Institute Foundation (Milano, Italy - permit number INT_18_2009). All efforts were deployed to minimize animal suffering.

### Human tumor xenografts, tumor vascularity and necrosis

Six- to eight-week-old female NOD/SCID mice with body weights of 20 to 25 g were purchased from Charles River (Milano, Italy, EU). The mice were housed under standard laboratory conditions according to our institutional guidelines. The human multiple myeloma KMS-11 cell line [19] was xenografted in nonobese diabetic/severe combined immunodeficient (NOD/SCID) mice as previously reported [17], [20]. Briefly, KMS-11 cells (5×10^6^ cells/mouse) were inoculated subcutaneously (SC) in the left flank of each mouse. When tumors were palpable (usually 10–12 days after tumor inoculation), nodules were already vascularized and showed a diameter of 7–10 mm with a weight of 0.210±0.070 g on average. At that time mice were randomly assigned to receive a 5-day course of either intraperitoneal (IP) sorafenib (90 mg/kg/day), oral sunitinib (40 mg/kg/day) or a single IP injection of 50 mg/kg combretastatin-A4-phosphate (CA4P) 72 hours before euthanasia. In these conditions vessels were already formed and could be targeted by antivascular agents. At the same time tumor were still growing, and tumor angiogenesis was still an active process that could be inhibited by antiangiogenic agents. Therefore both degree of vascularization and growth rate of the model were adequate to test antivascular or antiangiogenic compounds. Treatment protocols were adapted from the literature [21], [22], [23] in order to minimize differences in time course and to analyze all tumors at the same time after implantation. As reported, we performed a 5-day course treatment for antiangiogenic drugs whereas we slightly modified CA4P protocols performing a single injection of 50 mg/Kg CA4P 3 days before tumor evaluation. In our conditions we could observe necrotic effects in all samples, proving the action of the drugs, and at the same time spare still enough viable perinecrotic tissue to allow for a quantitative description. Efficiency of treatments were tested as described below and effects illustrated in the Results section.

Vessel walls were biotinylated *in vivo* through the intravenous injection of sulfosuccinimidyl-6-(biotinamido) hexanoate (sulfo-NHS-LC-biotin, Thermo Fisher Scientific, Rockford, IL, USA) as previously described [17], [20], [24]. Biotinylated tumors were then excised, fixed in 4% paraformaldehyde overnight at 4°C and embedded in 6% agarose. Agarose-embedded tumors were cut at a thickness of 100 µm using a vibratome, and the sections were stained with Alexa Fluor 488-conjugated streptavidin (Invitrogen, Carlsbad, CA, USA). To evaluate tumor morphology and necrosis, the same tumor samples were embedded in paraffin, cut to a 2 µm thickness and stained with hematoxylin and eosin. The sections were then examined as previously reported [20].

### Image sampling, acquisition and pre-processing

**Figure 1:**
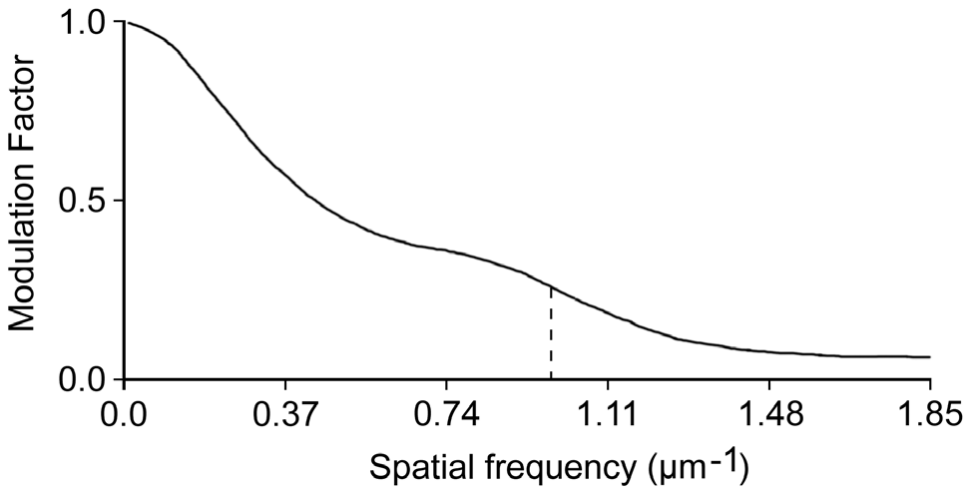
Spatial resolution of the acquisition system. The plot of the modulation transfer factor was obtained after imaging of a sharp edge. The stippled line highlights the maximal contrast obtainable at a spatial frequency of 1 µm^−1^ (0.54 cycles/pixel). The curve reports the feasibility to resolve details up to micrometric precision.

For each treatment, images were acquired from samples obtained from at least 2 independent experiments with at least 2 animals. For each tumor nodule, at least 2 z-series of vascular fields from 2 different 100 µm-thick sections were acquired using a MRC-1024 BioRad confocal microscope (BioRad Laboratories Inc., UK) equipped with a 40× oil immersion objective (NA 1.0) at lambda 488. Pixel dimension was calculated by the accompanying LaserSharp (BioRad Laboratories Inc., UK) software and resulted in a size of 0.54 µm. In the vertical axis, slices were 1 µm apart. The spatial resolution of the system was assessed imaging the sharp edge of a metal lamina in a fluorescent background. The image was then used to plot the correspondent Modulation Transfer Function (Figure 1) [25] using the imageJ program and the SE_MTF_2xNyquist plugin developed by Mitja *et al.* (http://rsbweb.nih.gov/ij/plugins/se-mtf/index.html). After sample acquisition, we converted the resulting 8-bit image stacks to isotropic stacks using the ImageJ software plug-in "Make Isotropic," which was developed by Cooper (http://rsbweb.nih.gov/ij/plugins/make-isotropic/index.html). The image contrast was then enhanced using a custom routine that normalized each slice when the dynamic range of the slice was greater than 30 grey levels. Finally, the stacks were converted to binary by applying a Renyi Entropy filter. This algorithm was selected from those available in ImageJ (http://rsb.info.nih.gov/ij/) because it minimized the detection of random noise and its results were among the closest to the thresholds chosen by a trained operator (see Supporting Information S1).

### Voxel classifications according to the size of vascular cross-sections through Cartesian planes

Binary voxels were assigned to different caliber vascular components based on their cross-sectional area and according to the workflow reported in Supporting Information S2. As a first step, we removed small, isolated particles (cross-sections smaller than 1 µm^2^) using an automatic 3D filter based on the TransformJ package [26], which we implemented as an ImageJ macro. (See Supporting Information S3). After filling in the hollow vessels, we classified the resulting voxels according to the minimum dimension of the cross-sections passing through the voxel that was calculated for one of the 3 Cartesian planes (see Supporting Information S4). Finally, these new caliber-filtered vessel maps were intersected with the input binary volume to obtain the final classification of input voxels. For each class of projected cross-section, we also calculated the volume ascribed to the lumen of hollow vessels by subtracting the unfilled vessels from the filled volumes.

### Surface rendering and vessel skeletonization

Color renderings of treated and untreated vascular networks were obtained following recovery of the original voxel intensities by intersecting 8-bit greyscale stacks with the binary volumes of mapped vessels of a defined caliber. Next, the new images were color-coded, according to their projected cross-sectional area, as small (1–5 µm^2^; cyan), medium (5–19 µm^2^; yellow) and large (19–75 µm^2^; red) and were merged together in a RGB stack. Finally, we visualized the resulting stack with the ImageJ 3D viewer rendering tool.

Stack skeletonization was performed using the Skeletonize (2D/3D) ImageJ plug-in (http://imagejdocu.tudor.lu/doku.php?id=plugin:morphology:skeletonize3d:start), which was developed by Arganda-Carreras and based on the decision tree algorithm created by Lee, et al. [27].

### Analysis of percent volume and spatial distribution

The percent volume of a sample was calculated as the ratio of all black voxels to the total voxels of the volume. Then, to calculate the spatial distribution, we used a 3D extension of a procedure we have previously described [17]. According to the original 2D procedure it is possible to discriminate between homogeneous or clusterized distributions of pixels in an area. This step is achieved dilating pixels by mathematical morphology operators until the expanding surface occupy a pre-defined fraction (usually 90%) of the area itself. When necessary, a normalization step allows to compare distributions in equal surfaces showing different amount of pixels. To this purpose, the pixels in each area are firstly expanded until they occupy an equal, minimal fraction of the surface. Then their distribution can be calculated.

In the present work we have adapted this procedure to the 3D analysis of volumes. Briefly, each volume was filled up to 90% with expanding black voxels in 3D using a rhombicuboctahedral dilation (Supporting Information S5), which was obtained by alternating the mathematical morphology operators 3D-cross and cuboidal dilation [28]. The final number of dilation cycles needed to fill exactly 90% of the volume was inferred by linear interpolation considering the nearest results encompassing this value. This number was dubbed volumetric Halo index (Hv) by analogy with the planar Halo index we already described [17]. For each analysis, the greatest initial volume observed among all of the volumes to be analyzed was used as the normalization value. In this respect, for each sample, the number of initial dilation cycles needed to reach the normalization value was subtracted from the total number of cycles needed to fill 90% of the volume to give a normalized, volumetric Halo index (nHv). According to the protocol of analysis, higher nHv numbers correlated with poorer volume-filling layouts, i.e. more clusterized distributions. This approach was implemented both as an ImageJ plugin and as a macro and was validated against artificial and real voxel distributions (Supporting Information S6).

### Software and statistical analyses

The main ImageJ scripts used in this study and the ImageJ plugin are provided as supplementary information files together with instructions for use (Supporting Information S7). Document Supporting Information S8 details the code for a 3D filter (needs the TransformJ package [26]). Document Supporting Information S9 present the source of the macro performing voxel classification according to the minimal Cartesian surface to which they belong. For spatial dispersion refer to documents Supporting Information S10 (source code of plugin) or Supporting Information S11 (macro). Routines can be tested with data from file Supporting Information S12 which contains 2 images referring to a control tumor stack at different steps during processing. ImageJ routines and all images of tumor vessels, both treated or not, have also been deposited in the Dryad repository 'http://dx.doi.org/10.5061/dryad.6c44q.

Analyses and tests were performed using the R software environment [29] and its basic statistical package. In descriptive statistics, medians were preferred to mean values because they are more robust statistical indicators. Bivariate data dispersion around the median values was generally indicated with the 25–75% interquartile range because the distribution of the nHv values diverged from normality. All data obtained from automatic routines are reported in spreadsheets Supporting Information S13 and Supporting Information S14 for reader's convenience. Curve fitting and the relative coefficient of determination (R^2^) were obtained using the curve fitter tool in ImageJ.

## Results

### Effects of vascular-targeted therapies

**Figure 2:**
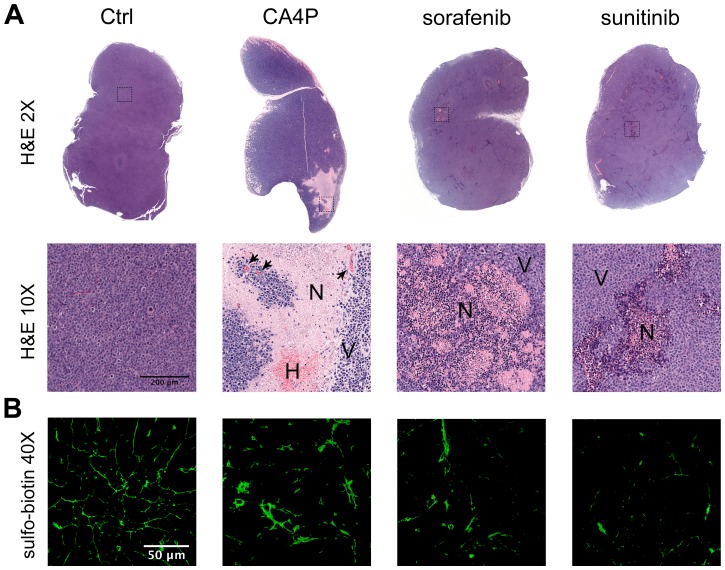
Effects of antiangiogenic and antivascular treatments on KMS-11 tumor nodules. Panel A. Biotinylated tumor samples for 3D vascular analysis were embedded in paraffin, cut to a 2 µm thickness and stained with hematoxylin and eosin to reveal necrotic areas. Representative images are shown. Objective lens, original magnification: 0.08 NA dry objective, 2× and 0.4 NA dry objective, 10x. N, necrosis; V, viable cells; H, haemorrhagic areas. Arrows indicate congested vessels. Panel B. Biotinylated vessels were revealed by staining sections with Alexa Fluor 488-conjugated streptavidin. Representative 2D images of tumor samples for 3D vascular analysis are shown. Objective lens, original magnification: 1.0 NA oil objective, 40x.

As a preliminary control, we evaluated whether the three drugs used in this study, including the antiangiogenic agents sorafenib [30] and sunitinib [31], [32] and the vascular-disrupting agent CA4P [33], [34], caused a significant increase in the number of necrotic areas in KMS-11 tumor nodules (Figure 2) as compared to controls. In particular, sorafenib and sunitinib caused widespread and scattered areas of necrosis within tumor nodules and a strong reduction in tumor vascularization [30], [32]. In contrast, CA4P-treated tumors presented massive necrotic areas associated with hemorrhagic spots and congested and coagulated vessels [33].

### Identification of microvessels and classification according to cross-section

We identified vessel walls by applying the Renyi Entropy threshold algorithm to isotropic, contrast-enhanced, grey-scale stacks acquired from sulfo-biotin-stained samples. This simple approach provided clean volumes devoid of random noise but other procedures can be used to obtain binary representations of tumor vessel networks [35]. For each binary volume, identified voxels were divided among several stacks in agreement with the approximate cross-sectional area of the microvessel they represented. However, to address the presence of voxels that belonged to a vessel but were not connected to it, we assigned voxels to vessels according to a reconstructed map of filled microvessels (Supporting Information S2 and S4) rather than considering voxels aside from their context.

**Figure 3:**
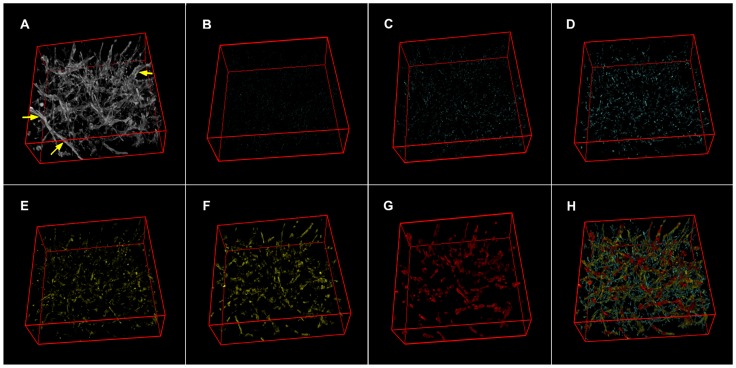
Example of vessel classification according to the approximate size of the cross-section. A. Surface rendering of an 8-bit stack representing a vascular bed from an untreated KMS-11 nodule. B to G. Surface renderings of filtered vessels classified using the first set of bins listed in Table 1. Vascular components were arbitrarily color-coded, according to their cross-sectional area, as small (1–5 µm^2^; cyan), medium (5–19 µm^2^; yellow) and large (19–75 µm^2^; red) H. Surface rendering of the reconstituted organization of filtered vessels; the volumes of B to G were summed while preserving their colors. Arrowheads in panel A point to vessels efficiently removed from panel H due to exceedingly large cross-sections.

Microvessels, which demonstrated approximate cross-sectional areas ranging from 1 to 75 µm^2^ (less than 10 µm diameters for cylindrical vessels), were classified using 4 redundant sets of bins in geometric sequence (measures in µm^2^). First set 1–2, 2–5, 5–9, 9–19, 19–37, 37–75 see Table 1, second set 1–3, 3–6, 6–12, 12–23, 23–47, third set 2–3, 3–7, 7–14, 14–28, 28–56 fourth set 2–4, 4–8, 8–16, 16–33, 33–65. This choice reflected our desire to use a large number of classes without increasing misclassification due to poor bin size. In fact, under the conditions applied, the measure of the cross-sectional surface S of a vessel could not be precisely assessed from the value of its minimal projection on Cartesian planes (P). Depending from vessel orientation, S could range from P to P√2 increasing unreliability of classification along with reduction in bin size. An example of the results of the classification process is shown in Figure 3 using the set of bins from Table 1 on the vessels shown in panel A. As an additional effect, the procedure efficiently removed vessels with cross-sections larger than the upper limit of the set (arrowheads in panel 3A).

**Table 1:** Details of vessel classification.

Lower limit - Upper limit (px)	Projected vessel cross-section ( µm^2^)	Symbols
4 – 8	1 – 2	Open diamond
8 – 16	2 – 5	Closed diamond
16 – 32	5 – 9	Open triangle
32 – 64	9 – 19	Closed triangle
64 – 128	19 – 37	Open circle
128 – 256	37 – 75	Closed circle

**Figure 4:**
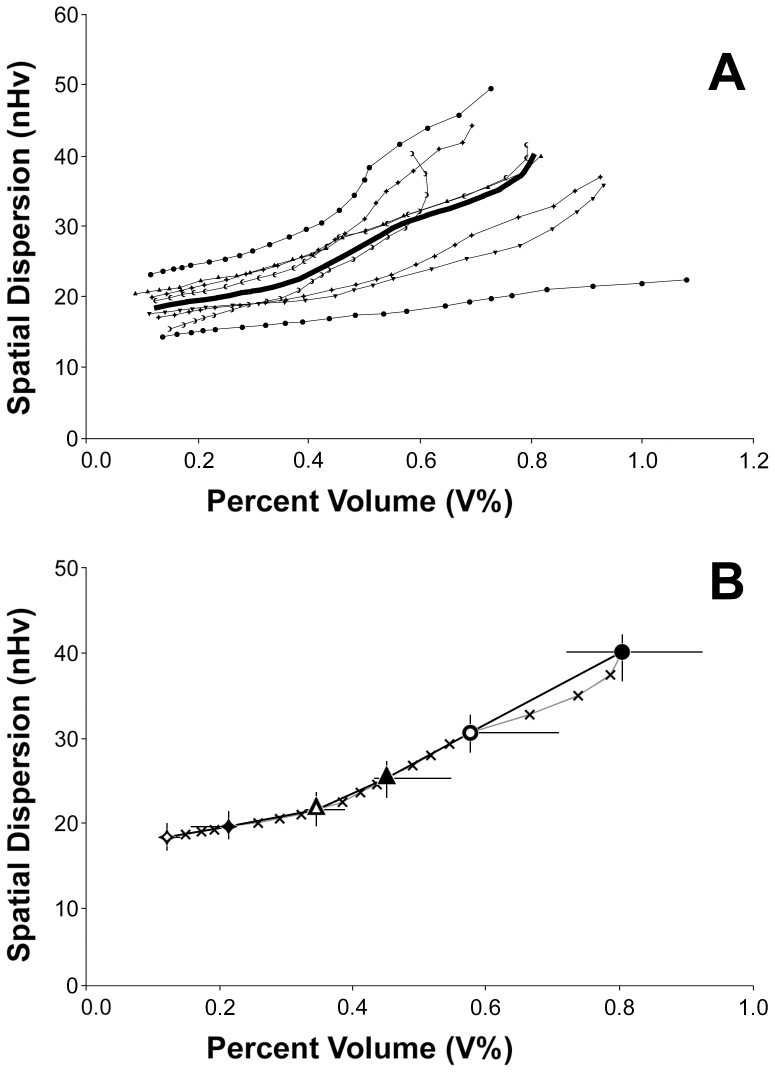
Z-projection plot of untreated KMS-11 tumor nodules analyzed in terms of staining amount and dispersion. Spatial dispersion is expressed in terms of normalized volumetric Halo values (nHv). Panel A. Symbols identify 8 different samples containing vessels that were classified according to cross-section from the 21 classes listed in Table 1. Identical symbols refer to results obtained from the same sample and are shown connected with a line to demonstrate their relationship. The large black line connects the medians obtained from all the classes of samples. Panel B. Open and closed diamonds, triangles and circles represent median values from the same 8 samples shown in A, which contained vessels that were classified according to the first set of classes reported in Table 1. Crosses represent median values after vessel classification according to all of the 21 classes (identical to the black curve in panel A). For the black symbols, horizontal and vertical bars mark the IQR 50 (25–75%) of the samples.

**Figure 5:**
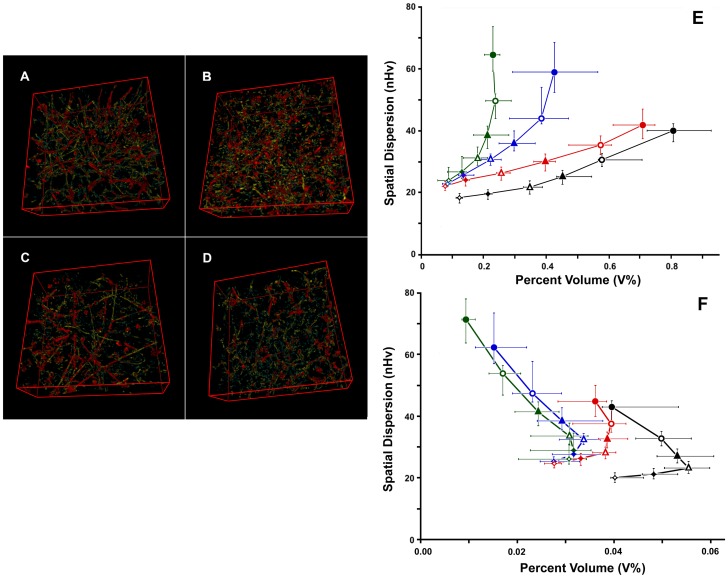
Effects of treatments on vessel quantification. Panels A to D. Color-coded renderings of vascular trees from untreated and treated samples. Vessels are shown grouped according to their approximate cross-section area as small (1–5 µm^2^; cyan), medium (5–19 µm^2^; yellow) and large (19–75 µm^2^; red) caliber vessels. A, untreated KMS-11 nodules; B, CA4P; C, sorafenib; D, sunitinib. Panel E. z-projection plot of all of the 24 filtered stacks for the 4 treatments: black, untreated; red, CA4P; blue, sorafenib; green, sunitinb. Open and closed diamonds, triangles and circles refer, in order, to classes of vessels of increasing caliber (see first set in Table 1). Spatial dispersion is expressed in terms of normalized volumetric Halo values (nHv). The chart reports the medians and IQR 50, for both percent volume and spatial dispersion from 8 samples. Inside the same treatment, points relative to vessels with increasing caliber are connected with a line of the appropriate color. Panel F is similar to E, although the data were obtained after vessel skeletonization to remove the contribution of wall thickness.

**Figure 6:**
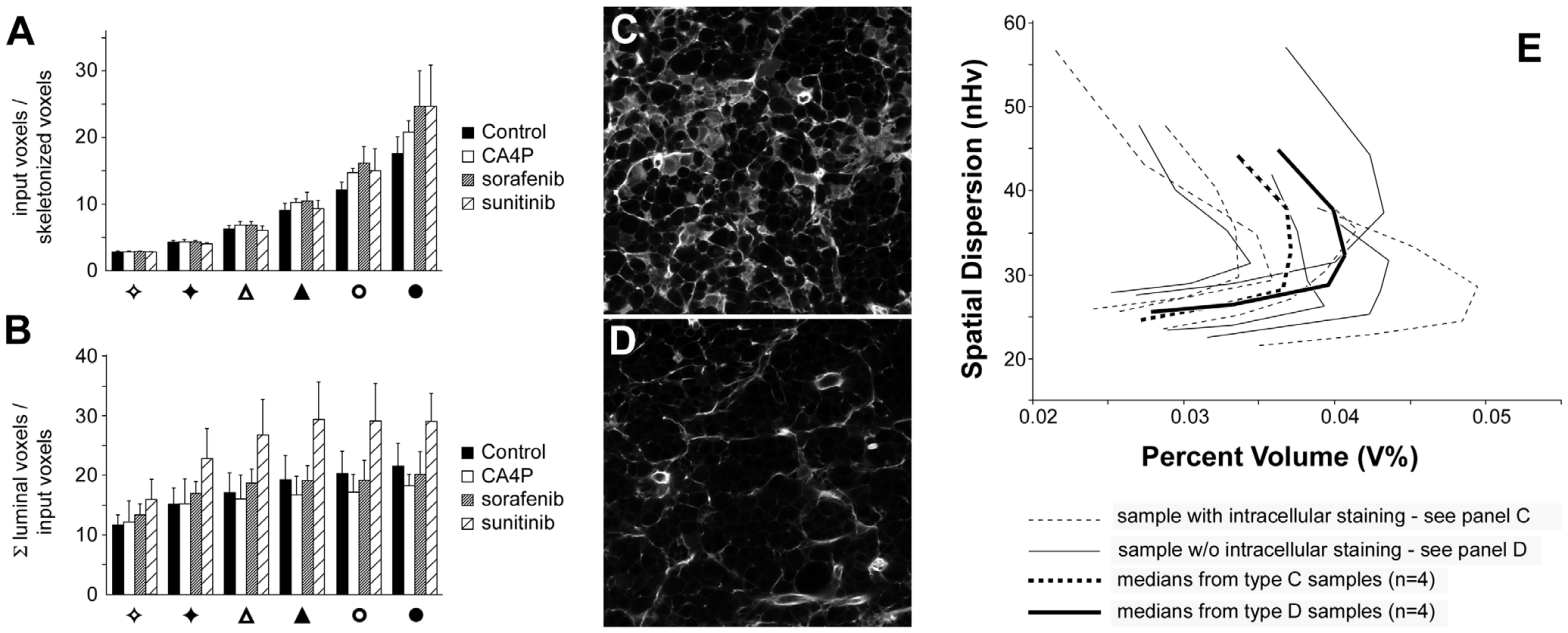
Exclusion of artifacts in CA4P-treated vessels. Input voxels were obtained after image thresholding by removing very small particles (see Figure S2). Panel A. Search for CA4P-specific changes in the ratio between the amounts of input binary voxels and skeletonized voxels. Ratios are reported on the Y axis. Symbols group results from vessels classified in the 6 bins of Table 1. Error bars refer to standard deviations, n = 8. Panel B. Search for CA4P-specific changes in the ratio between total luminal volume and input voxels. Symbols and bars as in panel A. Error bars refer to standard deviations, n = 8. Panels C and D. Representative images of CA4P-treated samples showing intracellular staining (C) or not (D). Panel E. z-projection plot of CA4P-treated samples divided in 2 groups (n = 4) according to the presence of intracellular staining. Spatial dispersion is expressed in terms of normalized volumetric Halo values (nHv). Dotted lines, samples with intracellular staining; solid lines, normal staining. The thin curves refer to data from individual samples, whereas the large black curves connect medians for the percent volume and spatial dispersion.

The table reports the parameters and limits (pixels) of the first set of bins used to classify vessels, as well as the value range of the projected relative cross-sections. In addition column 3 lists the symbols used to indicate the relative results plotted in Figures 4, 5 and 6.

### An unexpected relationship links the amount, dispersion and caliber of microvessels

For all of the classes that were used to divide the vascular components of the analyzed vascular networks, we calculated the percent of voxels (V%) and a normalized volumetric Halo value (nHv) reflecting the spatial distribution of the voxels themselves.

An analysis of the stacks derived from untreated KMS-11 nodules is shown in Figure 4 as a z-projection of a 3D plot with the classes of vessels ordered along the *z*-axis and the values for V% and nHv oriented along the abscissa and the ordinate, respectively. Surprisingly, the points resulting from the analysis were mainly organized in monotonic sequences (Figure 4A) and were neither scattered all over the plot nor grouped in a restricted area. This organization was confirmed for each sample by fitting all of the points with a 2^nd^ degree polynomial curve. The goodness of fit was confirmed according to the values of the coefficient of determination (R^2^), which was consistently greater than 0.93 and was even greater than 0.98 for 7 of the 8 curves. Thus, these findings demonstrated the existence of an unexpected relationship between vessel wall quantity, spatial distribution and caliber. Interestingly, the results from the analysis of vessels that were classified as a single set of non-overlapping classes (Figure 4B) were sufficient to approximate the organization of all of the points in the plot, which eliminated the need to classify vessels in a redundant way. Consequently, the remaining results were collected from vessels that were classified using only the first set of bins, as reported in Table 1.

### Vascular-targeted therapies alter the relationship between microvessel quantity, distribution and caliber

The analysis performed on the controls was repeated on tumors treated with vascular-targeted drugs, including the antiangiogenic agents sorafenib [30] and sunitinib [31], [32] and the vascular-disrupting agent CA4P [33], [34]. Figure 5A to D shows still images from color renderings of the vascular networks in the analysis to highlight differences in network organization. In addition, Figure 5E reveals the results of the analyses, as shown as a z-projection of the plot between the projected cross-sectional values and the values of V% and nHv.

All vascular-targeted therapies induced changes with respect to the controls, and these changes were markedly evident for the antiangiogenic agents that caused a general reduction in the quantity of vessels and a corresponding heterogeneous distribution of the vessels. Moreover, this effect increased with increasing vessel cross-sectional areas and reached a maximum effect for the thickest microvessels examined.

In contrast, when tumors were treated with the antivascular drug CA4P, only thin microvessels appeared to show a reduction in percent volume and space-filling values. Thus, these findings demonstrated the specific actions of each of the 3 therapies applied.

### Removing effects due to wall staining

To allow for an improved comparison between the effects of different therapies, we removed variability due to treatment-specific changes in vessel staining. To do so, filled vessels were reduced to their basic skeletons using the ImageJ plug-in Skeletonize (2D/3D). Skeletonized voxels could then be classified into different stacks according to the size of the cross-section, which was calculated on the vascular maps that were previously used to classify input voxels.

An analysis of the volume and distribution of skeletonized vessels is reported in Figure 5F. Under these new conditions, vessels treated with sorafenib or sunitinib produced similar values, which were markedly different from the controls. Similarly, results obtained from CA4P-treated vessels mapped to a different area of the plot with respect to the controls and vessels treated with antiangiogenic drugs. Spatial differences were tested for statistical significance using Welch's 2-tailed *t*-test and Wilcoxon rank tests, and the results are shown in Table 2. With respect to the controls, the loss of vessels induced by CA4P consistently appeared significant (p<0.05), as significant changes were observed regarding the spatial distribution of vessels in sections with cross-sectional areas as large as 19 µm^2^. In contrast, nearly all of the differences observed between the controls and sorafenib- or sunitinb-treated samples were highly significant (p<0.001), both in volume and spatial distribution.

**Table 2:** Significance of observed differences.

		Projected vessel cross-section ( µm^2^)																	
	S	1 – 2			2 – 5			5 – 9			9–19			19–37			37–75		
		C	CP	Sf	C	CP	Sf	C	CP	Sf	C	CP	Sf	C	CP	Sf	C	CP	Sf
Δ Vol%	CP	***			***			***			**			**			*		
	Sf	**	ns		**	ns		***	ns		***	ns		***	**		***	**	
	Sn	**	ns	ns	***	ns	ns	***	*	ns	***	***	*	***	***	ns	***	***	*
Δ nHv 90%	CP	**			**			*			*			ns			ns		
	Sf	**	ns		***	ns		***	*		***	*		***	**		**	**	
	Sn	**	ns	ns	***	ns	ns	***	*	ns	***	**	ns	***	***	ns	***	***	ns

Summary of statistical tests between Vol% (&delta Vol% - Welch's t-test) and nHv 90% (&delta nH 90% - Wilcoxon rank test) for all of the classes of cross-sections analyzed and shown in Figure 5. S, cross-section; C, controls; CP, CA4P; Sf, sorafenib; Sn, sunitinib. Significance: ns, not significant; *, p<0.05; **, p<0.01; ***, p<0.001.

### Exclusion of possible artifacts induced by CA4P treatment

Vascular-disrupting agents (VDA) may alter the apparent dimension of vessels after sulfo-biotin staining as a consequence of dye uptake by damaged endothelial cells [33], [36]. To evaluate this possibility, which could artificially alter the results even after skeletonization, we followed 3 different approaches. First, remembering that input voxels were obtained after image thresholding by removing very small particles (see Figure S2), we assessed the ratio between the amount of input voxels and the amount of skeletonized voxels. Given that the number of skeletonized voxels is influenced by the length of the vessels, this ratio was roughly the average amount of sulfo-biotin binding per unit of vessel length. Treatments should not alter this value; however, in the event of dye uptake by cells we expected a marked increase because of apparently thicker cell walls. Figure 6A shows that treatment with CA4P increased this value less than treatments with sorafenib and sunitinib, although the overall effect was marginal and observed only for the largest vessels.

Next, we assessed the ratio between the total luminal volume of the vessels and the corresponding quantity of the input voxels (Figure 6B). This parameter is inversely related to wall thickness and in the case of artefactual cell staining we expected a drop in its value. However, data indicated that CA4P samples presented similar results as untreated nodules for most classes of cross-section with only a slight reduction for large vessels. Samples treated with sorafenib behaved as controls whereas vessels treated with sunitinib presented a significant increase (p<0.05, n = 8) in this ratio when vessels with cross-sectional areas greater than 2 µm^2^ were analyzed. These results suggested that sunitinb increased the quantity of hollow vessels or at least their roundness.

Finally, for CA4P samples, we performed a direct comparison. Given that half of our CA4P samples presented sulfo-biotin-stained cell bodies before vessel identification (Figures 6C and 6D), we divided the 8 CA4P stacks into 2 groups (n = 4), one presenting stained cell bodies and one presenting normal vessel staining. Figure 6E shows the results obtained by comparing the skeletonized vessels from these groups; the plot displays similar curves and comparable values for percent volume and spatial distribution. All together these results proved that the stained cell bodies observed in greyscale images were correctly excluded from binary images, minimizing the presence of artifacts in CA4P samples. In addition, the similar shape of the curves in Figure 6E excluded also the potential misclassification of vessels due to artificially increased cross-sectional areas.

## Discussion

In this work, we report the development of an approach used to characterize tumor vascular networks according to microvessel length, distribution and caliber. This analysis was restricted to perinecrotic microvessels with a maximum approximate cross-sectional area of 75 µm^2^ (about 10 µm in diameter for cylindrical vessels), which enabled the characterization of different qualitative and quantitative effects of antiangiogenic or antivascular drugs on human tumor xenografts.

Unlike other efforts that have addressed the morphological features of vascular trees [11], [12], [13] or their underlying physical characteristics [14], our approach attempted to identify changes in the vascular bed related to the arrangement of its small capillaries. Two lines of thought prompted us to focus on these structures. First, the presence of a microvessel in a given location reflects the possibility for that tissutal environment to produce or maintain a vessel of that caliber. Thus, by utilizing a tumor model in which microvessels are evenly distributed throughout the tissue, we sought to use these microvessels as probes to detect even small changes in tumor vascularization. Second, small microvessels, as compared to larger vessels, are fundamental to the oxygen and nutrient distribution throughout the tissue [18], which suggests that we should be able to correlate changes at this level with changes in tissue viability.

In the course of our analysis, we observed the existence of a relationship between vessel quantity and dispersion when considering vessels of different calibers. Given that the data from each vessel class concur in regards to the dynamic balance between vessel formation and loss at a given time in a given tissue location, the meaning of this relationship appears related to the efficiency of vascularization of the tissue, *i.e.*, the ability to sustain the growth of vessels from initial sprouts into functional capillaries. Thus, following treatment with sorafenib or sunitinib, changes in the ratio between ultrathin, immature stalks and RBC-conveying capillaries highlighted the expected blockade of tumor angiogenesis.

Concerns can be expressed about the determination of vessel dimensions following our approach and really several factors contribute to spoil a precise measurement of the cross-sectional area of the vessels. In spite that our samples were analyzed by optical sectioning of a thick slice, we cannot rule out alterations due to tissue shrinkage in the phase of sample preparation. In this respect, other methods such as synchrotron radiation-based micro computed tomography (SRµCT) [37] both in absorption [38] and phase contrast modes [39] can provide high-resolution imaging of 3D vascular networks. Moreover, phase contrast SRµCT has been recently shown to minimize sample alterations not only avoiding sectioning and shrinkage but also bypassing the use of contrast or casting agents needed in absorption µCT [39]. Thus, SRµCT provides advantages over confocal analysis in the trustworthy determination of 3D vascular networks. Unfortunately, the need for a synchrotron facility limits its overall availability.

Along with tissue shrinkage, measurement of vessel caliber by projection on a Cartesian plane introduced an additional uncertainty. According to vessels orientation, vessel calibers can be underestimated, their real values being sometimes larger up to 1.41 times. Thus vessel calibers should be regarded only as indicative values. This inaccuracy prevented us to derive meaningful diameter estimates given also that we were unable to exclude changes in cross-section shapes due to drug-specific effects. Despite these evident drawbacks, comparisons between treatments were not affected by possible misclassification of the cross-sections of vessels, given that all samples from different treatments were classified by the same automatic procedure, treatments did not selectively alter orientation and we analyzed large numbers of samples.

Although both antiangiogenic treatments demonstrated similar effects on skeletonized vessels, we were able to discriminate between these effects based on the luminal volume of the vessels. These differences were not due to a reduction in the level of sulfo-biotin binding, but may have been linked to the specific effects of the drugs [40], [41], [42], [43]. In this respect, sunitinib is known to destabilize vessels through the loss of pericyte coverage [44], [45] as a consequence of the inhibition of PDGFR signaling [41]. Although this loss has been correlated with the increase in metastatic potential observed following sunitinib treatment [46], the current study did not address this point, and further analyses are required to link the differences we observed to any biological effect.

The results of treatment with the antivascular drug CA4P presented a reduction of RBC-conveying capillaries together with an increased loss of thinner microvessels and ultrathin vessel sprouts. However, these effects did not affect the shape and orientation of the relationship between microvessels of different caliber, which remained close to the control values, despite the presence of large necrotic areas in the samples. Given that we were able to rule out the presence of artefacts due to altered sulfo-biotin uptake by endothelial cells, these results demonstrate that the antivascular action of CA4P did not affect the mechanisms of tumor vessel formation.

Together, these results demonstrate that this study was able to detail vascularization efficiency to an unprecedented extent, as well as the effects induced by vascular-targeted therapies on tumor vessels. In this respect, our efforts may provide an alternative rationale for the multifaceted characterization of physio-pathological vascular networks, at least at the microvessel level.

## Supporting Information

Supporting Information S1 Rationale for the choice of the algorithm used to convert 8-bit grayscale stacks into binary.
(PDF)

Supporting Information S2 Workflow of the analysis from the acquisition of confocal stacks to the skeletonization of binary vessels.
(PDF)

Supporting Information S3 Rationale for 3D filtering using the ImageJ analyze particle command and the TransformJ plug-in.
(PDF)

Supporting Information S4 Exemplification of vessel fill-up and voxel classification according to the smallest Cartesian cross-section to which they belong.
(PDF)

Supporting Information S5 Rationale for practical rhombicuboctahedral dilation using ImageJ.
(PDF)

Supporting Information S6 Validation of the imageJ informatics tools (*i.e.*, macros and plug-ins) used to calculate the number of cycles needed to fill 90% of a volume by rhombicuboctahedral dilation.
(PDF)

Supporting Information S7 Description and use of the informatic routines presented in this study.
(PDF)

Supporting Information S8 Text of an ImageJ macro used to remove small unconnected particles from samples before voxel classification.
(TXT)

Supporting Information S9 Text of an ImageJ macro used to classify voxels in a binary stack according to the minimal Cartesian surface to which they belong (*i.e.*, the approximated vessel cross-section).
(TXT)

Supporting Information S10 **Source code for the ImageJ plug-in used to calculate the 3D spatial dispersion of vessels in binary stacks.** Expansion was performed by rhombicuboctahedral dilation after volume normalization according to the total number of vessel voxels.
(JAVA)

Supporting Information S11 Text of an ImageJ macro performing as a plug-in.
(TXT)

Supporting Information S12 A compressed file containing a folder with 2 image files referring to different processing steps for an acquired image from an untreated KMS-11 tumor sample. The folder, that can be treated as a test data set, contains: a) Image binadjG12S_Ctrl1-iso.tif which is the binary image resulting from Renyi Entropy thresholding of an isotropic, contrast-enhanced, 8-bit stack ready to be turned binary. b) Image FOR4baG12S_Ctrl1-iso.tif which is the binary image after removal of particles with Cartesian sections less than 1 µm^2^ (4 px). This last image is ready to be splitted into subimages, according to vessel cross-sections, using macro Vessel_Calibrometry.txt.
(ZIP)

Supporting Information S13 List of the experimental values used to calculate and draw the graphs shown in the paper as Figure 4.
(XLS)

Supporting Information S14 List of the experimental values used to calculate and draw the graphs shown in the paper as Figures 5 and 6.
(XLS)
