# Correction: D Quantification of Tumor Vasculature in Lymphoma Xenografts in NOD/SCID Mice Allows to Detect Differences among Vascular-Targeted Therapies

**DOI:** 10.1371/annotation/f61040ca-4eab-4c4c-ad77-0a2ab8c503de

**Published:** 2013-04-03

**Authors:** Marco Righi, Arianna Giacomini, Loredana Cleris, Carmelo Carlo-Stella

There was an error in the title of the article. The correct title is:

3D Quantification of Tumor Vasculature in Lymphoma Xenografts in NOD/SCID Mice Allows to Detect Differences among Vascular-Targeted Therapies

The correct citation is:

Righi M, Giacomini A, Cleris L, Carlo-Stella C (2013) 3D Quantification of Tumor Vasculature in Lymphoma Xenografts in NOD/SCID Mice Allows to Detect Differences among Vascular-Targeted Therapies. PLoS ONE 8(3): e59691. doi:10.1371/journal.pone.0059691

