# Supplementary material for: D Quantification of Tumor Vasculature in Lymphoma Xenografts in NOD/SCID Mice Allows to Detect Differences among Vascular-Targeted Therapies
Source: PLoS One. 2013 Mar 26;8(3):e59691. doi: 10.1371/journal.pone.0059691 (PMC3608557; doi:10.1371/journal.pone.0059691)
Supplement: Supporting Information S1 — Rationale for the choice of the algorithm used to convert 8-bit grayscale stacks into binary. (PDF) [file pone.0059691.s001.pdf]

A

Threshold method	Algorithm - manual	Absolute (Algorithm - manual)
Default ImageJ	- 19.273 ± 5.875	26.039 ± 4.522
Maximum entropy	18.016 ± 16.543	27.906 ± 6.232
Renyi entropy	10.898 ± 19.163	28.523 ± 5.628
Shanbag	76.477 ± 17.170	80.070 ± 14.540
Yen	11.484 ± 20.927	31.313 ± 5.799

B

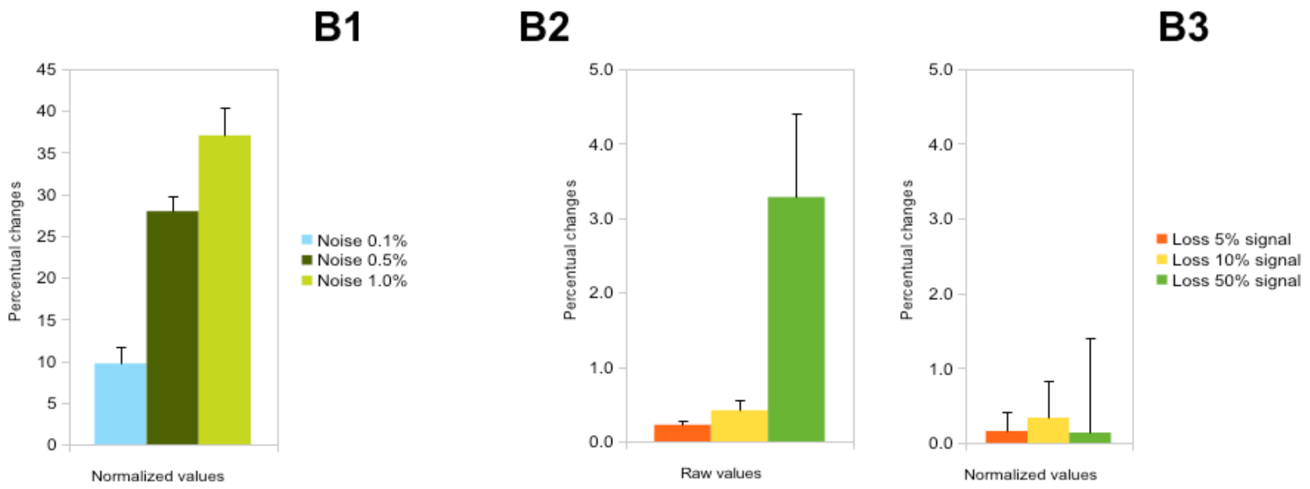

Figure S1. Panel A. Differences between thresholds obtained by different thresholding algorithms and those choosen by a trained operator on 128 slices from 4 groups of 2 independent 8-bit stacks acquired from untreated samples or from samples treated with combretastatin-4-phosphate (CA4P), sorafenib and sunitinib. The table reports the average values, and the absolute average values, of the differences ± standard deviation. The default, max entropy and Renyi entropy methods gave close results when considering the absolute values. However the Renyi entropy algorithm identified values closer to manual thresholds, whereas the default algorithm selected more signal than required. Given the influence of unwanted noise on values of spatial distributions (see below), the Renyi entropy algorithm

was selected for this analysis.

Panel B. Determining the influence of random noise on spatial distributions of signal. Given 4 untreated tumor samples, we derived, for each of them, new distribution of vascular signals after adding random voxels up to 0.1%, 0.5% and 1% of the total signal, observed in each sample. Then we calculated the spatial distribution values (nHv) of every stack and the percentual changes observed using the formula: (original distribution - noisy distribution)*100 / original distribution. As it can be seen in graph B1, addition of as low as 0.1% of random voxels changed the spatial distribution value of about 10%, whereas addition of 1% random noise falsed the value of the spatial distribution by more than 35%.

At the contrary, failure to identify vascular voxels did not seem so critical, in respect to the assessment of spatial distribution. in the same 4 tumor samples we removed increasing amounts of vascular voxels (5%, 10% or 50%) still in a random pattern. Graphs B2 and B3 reports the impact of those eliminations on the values of spatial distribution (Hv) before and after normalization of the results according to the maximum amount of observed voxels. It is easy to see that, in normalized conditions, removal of up to half the voxels had little impact on spatial distribution values with changes restricted to less than 1%.

Because of these results, we approached vessel recognition in our samples trading thourough identification with trustworthy identification, accepting only stained voxels with an intensity sufficiently high to represent genuine vascular walls.
