# Supplementary material for: D Quantification of Tumor Vasculature in Lymphoma Xenografts in NOD/SCID Mice Allows to Detect Differences among Vascular-Targeted Therapies
Source: PLoS One. 2013 Mar 26;8(3):e59691. doi: 10.1371/journal.pone.0059691 (PMC3608557; doi:10.1371/journal.pone.0059691)
Supplement: Supporting Information S2 — Workflow of the analysis from the acquisition of confocal stacks to the skeletonization of binary vessels. (PDF) [file pone.0059691.s002.pdf]

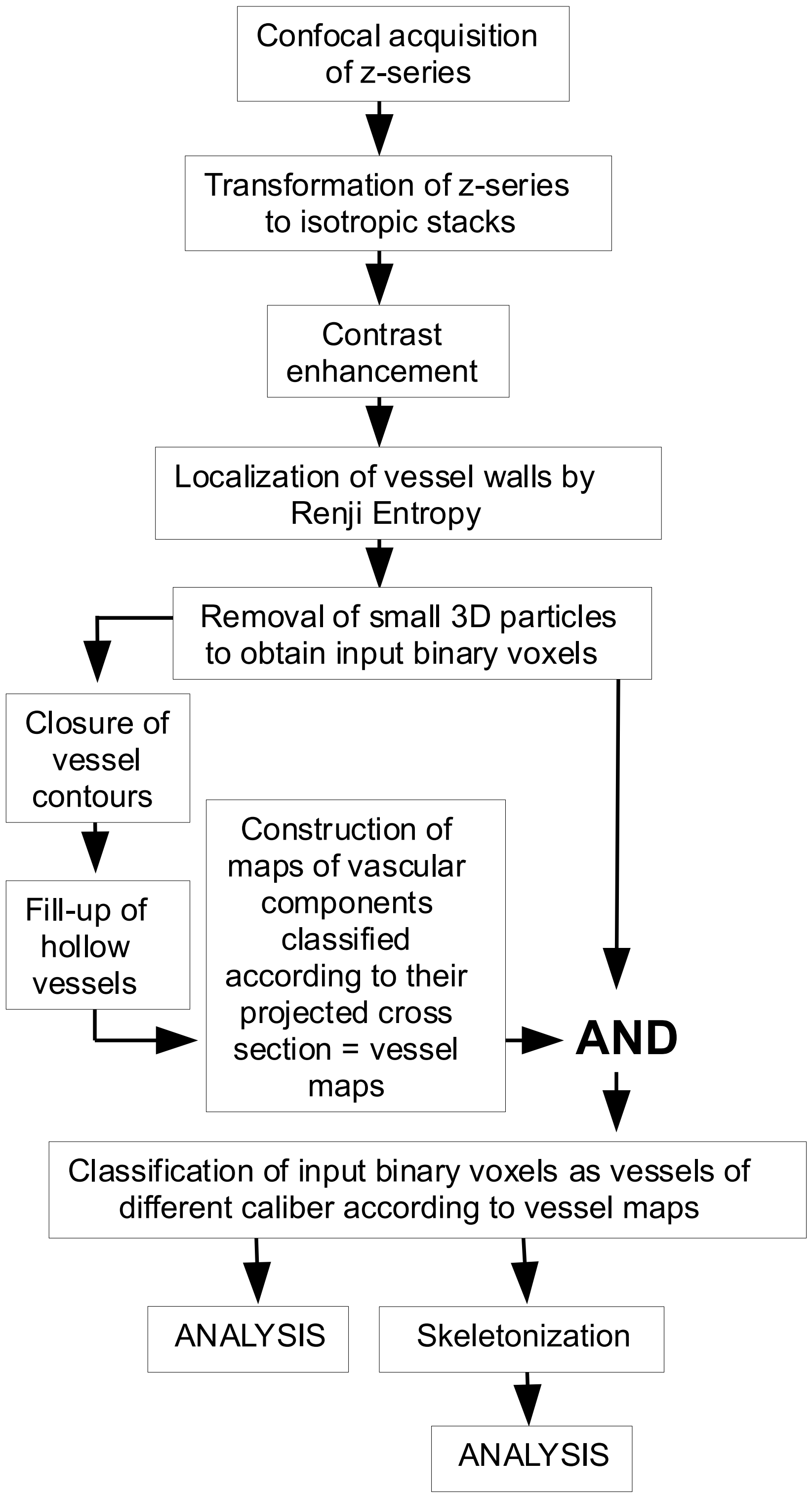

Figure S2. Workflow scheme for preparation of vessel maps and voxel classification according to intersection of vascular cross-sections with Cartesian planes. After classification, input voxels from vessels of different calibers were analyzed directly or following skeletonization.
