# Supplementary material for: D Quantification of Tumor Vasculature in Lymphoma Xenografts in NOD/SCID Mice Allows to Detect Differences among Vascular-Targeted Therapies
Source: PLoS One. 2013 Mar 26;8(3):e59691. doi: 10.1371/journal.pone.0059691 (PMC3608557; doi:10.1371/journal.pone.0059691)
Supplement: Supporting Information S3 — Rationale for 3D filtering using the ImageJ analyze particle command and the TransformJ plug-in. (PDF) [file pone.0059691.s003.pdf]

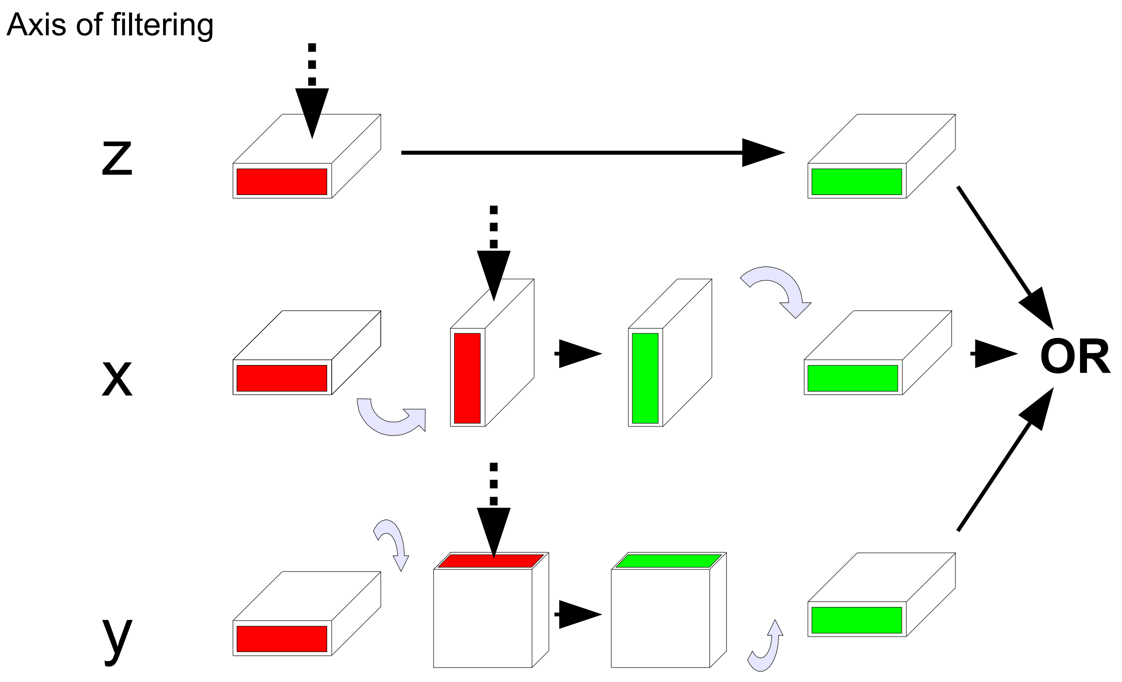

Figure S3. Removal of small isolated particles by means of a 3D filter. In order to protect our data against random noise we removed small isolated particles (unconnected to vessels) by means of a custom routine based on the ImageJ Analyze Particle command. Briefly, the routine started removing cross-sections lower than an arbitrary limit (1.2 μm^2 in this case), from each slice of the stack to be filtered (red volume) to obtain a new, z-filtered, stack (green volume, first line). Then (second line) the original stack was tilted by 90° around the y axis using the TransformJ package developed by Meijering *et al.* (see ref. 26 in the paper) and the procedure repeated, thus filtering along the x axis. At the end, the x filtered stack was tilted back to its original orientation. This tilting/filtering/tilting passage was repeated also for the y axis (third line) and the 3 filtered stacks (green volumes in right column) were combined by means of union operations (OR) giving a volume deprived of particles with cross-sections smaller than 1.2 μm^2, on each Cartesian plane.
