# Supplementary material for: D Quantification of Tumor Vasculature in Lymphoma Xenografts in NOD/SCID Mice Allows to Detect Differences among Vascular-Targeted Therapies
Source: PLoS One. 2013 Mar 26;8(3):e59691. doi: 10.1371/journal.pone.0059691 (PMC3608557; doi:10.1371/journal.pone.0059691)
Supplement: Supporting Information S4 — Exemplification of vessel fill-up and voxel classification according to the smallest Cartesian cross-section to which they belong. (PDF) [file pone.0059691.s004.pdf]

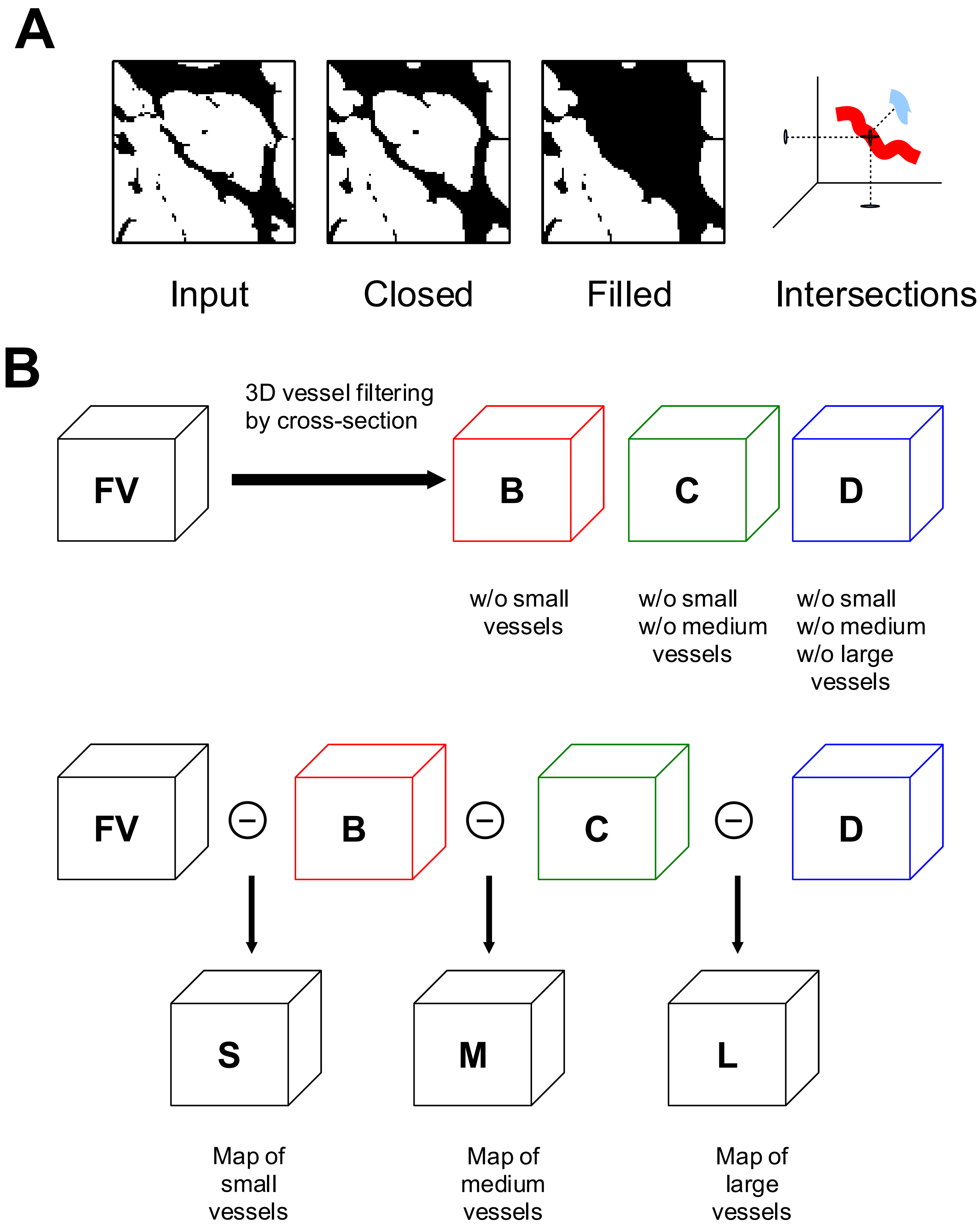


**Figure S4. Construction of vessel maps.** This procedure allowed to assign even isolated

voxels to vessels with larger cross sections without increasing the amount of signal observed in input volumes. **Panel A.** Filling hollow vessels. Most of the contours of poorly defined input vessels were closed by means of a mathematical morphology close operation with a 2 cycles dilation and erosion depth. Encircled spaces with an empty surface less than that of a specific threshold were filled, slice by slice, using the standard ImageJ "Fill holes" operation. The threshold was choosen to prevent the filling of holes with a surface of more than 350 $\mu m^2$, 4 times the cross section of the largest classified vessels, thus reducing artifacts due to the fill-up of lacunes delimited by connected vessels.

**Panel B.** Example of vessel classification. This step was performed on filled-vessel stacks using the same approach used to remove small isolated particles (see Figure S3) but combining the filtered stacks with a set of intersection operations (AND). This procedure granted that, for each vascular voxel, we could calculate the cross section of the vessel to which it belonged as the lowest value of the surface drawn on the 3 Cartesian planes containing the voxel iteslf. In this example, a filled-vessel volume (FV) was filtered according to the limits of 3 classification bins to give volumes B, C and D lacking respectively small vessels, small and medium vessels or small, medium and large vessels. Subsequently, the FV, B, C and D stacks were reused to obtain the localization maps of vessels with small, medium or large cross sections. Practically, we reused the filled-vessel stack as source to subtract the first filtered volume, lacking vessels with the smallest cross sections. This produced a map of the vessels with the smallest cross sections. We iterated the procedure now considering the former subtrahend volume as the new minuend volume, with the new subtrahend volume being the second filtered volume. These operations produced a set of stacks (dubbed vessel maps) which mapped the location of the filled vascular components with similar, minimal, Cartesian cross-section.

In the paper, the filtering operations were performed as in this example except that we used 6

classifying bins to obtain maps of vessels with approxymated cross-section comprised between the limits listed in the paper in Table I. Then, finally, these new, caliber-filtered, filled-vessel maps, were intersected with input binary voxels, to map them to specific stacks grouping voxels belonging to vessels of similar cross section.
