# Supplementary material for: D Quantification of Tumor Vasculature in Lymphoma Xenografts in NOD/SCID Mice Allows to Detect Differences among Vascular-Targeted Therapies
Source: PLoS One. 2013 Mar 26;8(3):e59691. doi: 10.1371/journal.pone.0059691 (PMC3608557; doi:10.1371/journal.pone.0059691)
Supplement: Supporting Information S5 — Rationale for practical rhombicuboctahedral dilation using ImageJ. (PDF) [file pone.0059691.s005.pdf]

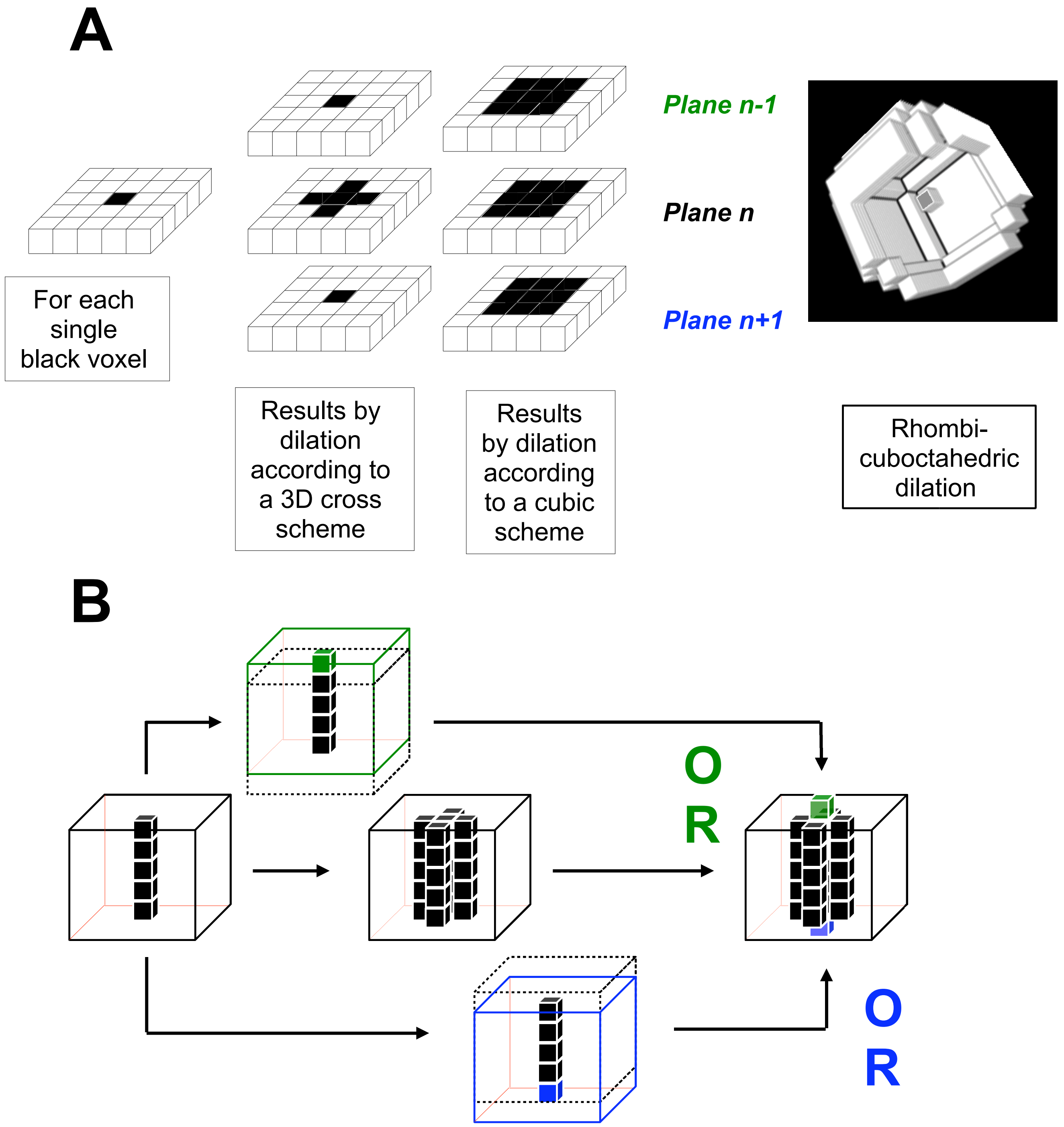

Figure S5. Rationale for 3D voxel dilation and space-filling measurement. Panel A. For each black voxel of a slice n of an image stack, 3D voxel dilation affected space occupancy in the n-1, n and n+1 planes, for both schemes of 3D-cross or cuboidal expansion.

Implementation as an ImageJ routine was possible considering that, in the case of 3D-cross dilation, contributes were equivalent to the input slice, for plane n-1 and n+1, whereas for plane n they summed up to a 4-connected planar dilation. At a difference, in the case of cuboidal expansion, contributes for all the planes were equivalent to a 8-connected planar dilation. Alternation of the 2 schemes produced, for a single voxel, a rhombicuboctahedral dilation (rightmost image) which best approxymated a spherical expansion.

Panel B. In the dilation of multivoxel samples, each expansion cycle produced 3 different volumes each collecting contributes for one class of planes originating from the different slices of the expanding stack. The final step of the cycle resolved in the union (OR) operation of the 3 volumes registered along the common portion, thus discarding out-of-volume contributes (green and blue voxels). Space occupancy was measured, cycle after cycle, as the ratio between black and total voxels of the stack until the fill-up of 90% of the volume (nHv 90%). Final nHv values were calculated by linear interpolation considering only 2 values encompassing the limit. These values were obtained every 2 alternated steps of dilation (a cuboidal dilation followed by a 3D cross voxel expansion) to base calculation only on results obtained by rhombicuboctahedral dilation.
