# Supplementary material for: D Quantification of Tumor Vasculature in Lymphoma Xenografts in NOD/SCID Mice Allows to Detect Differences among Vascular-Targeted Therapies
Source: PLoS One. 2013 Mar 26;8(3):e59691. doi: 10.1371/journal.pone.0059691 (PMC3608557; doi:10.1371/journal.pone.0059691)
Supplement: Supporting Information S6 — Validation of the imageJ informatics tools (i.e., macros and plug-ins) used to calculate the number of cycles needed to fill 90% of a volume by rhombicuboctahedral dilation. (PDF) [file pone.0059691.s006.pdf]

A

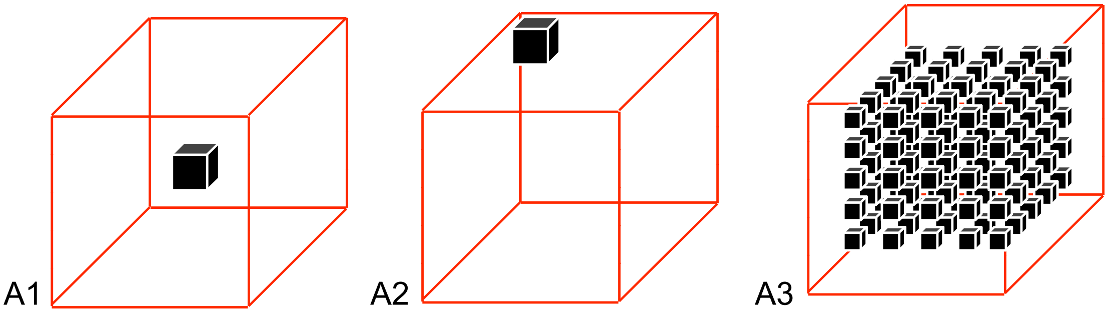

Voxels: 9x9x9 9x9x9 49x49x49

	Manual	ImageJ Macro	ImageJ Plugin
Volume A1	5.40247	5.40250	5.40246
Volume A2	9.56686	9.56690	9.56686
Volume A3	5.68878	5.68880	5.68877
Volume B1	ND	19.71170	19.71173
Volume B2	ND	19.37880	19.37884

B

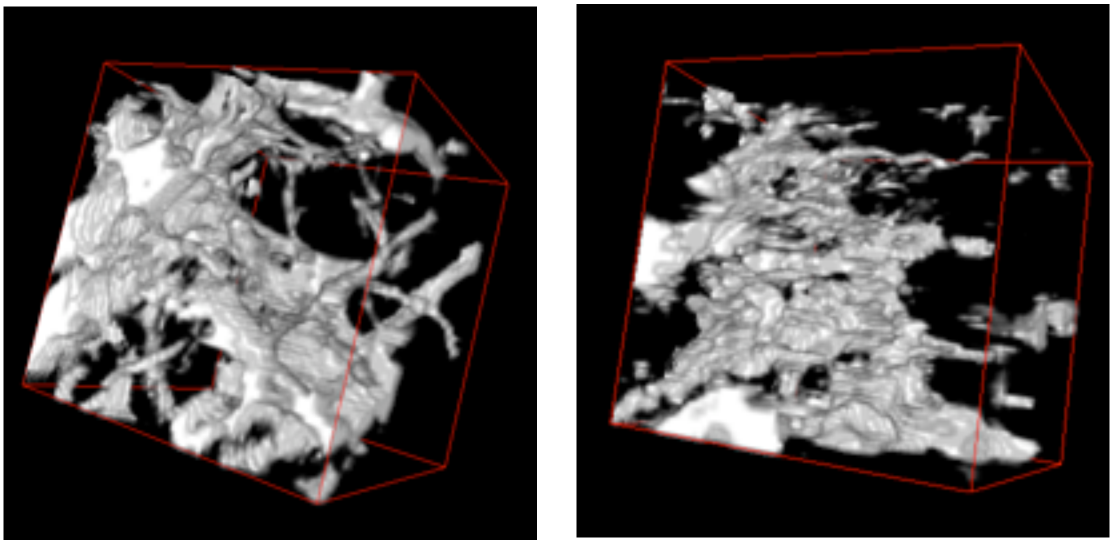

B1 B2

Voxels: 128x128x128 128x128x128

Figure S6. Panel A Validation of ImageJ routines for space-filling calculations on artificial point distributions. The values of nHv 90% for the 3 artificial point distributions shown were

calculated manually and then compared with those obtained with an ImageJ plugin and an ImageJ macro as internal control. Values, shown in the accompanying table, appeared identical if not for different levels of approxymation.

Panel B The values of the 2 additional binary distributions, shown as black and white renderings, were calculated by both the ImageJ plugin and the ImageJ macro, providing results differing only because of different levels of approxymation. This procedure was necessary because the ImageJ macro, whose operations could be followed visually in a step by step basis, served as a control of the results of the plugin on volumes whose nHv values could not be calculated manually.
