# Supplementary material for: D Quantification of Tumor Vasculature in Lymphoma Xenografts in NOD/SCID Mice Allows to Detect Differences among Vascular-Targeted Therapies
Source: PLoS One. 2013 Mar 26;8(3):e59691. doi: 10.1371/journal.pone.0059691 (PMC3608557; doi:10.1371/journal.pone.0059691)
Supplement: Supporting Information S7 — Description and use of the informatic routines presented in this study. (PDF) [file pone.0059691.s007.pdf]

3-D Quantification of Tumor Vasculature in Lymphoma Xenografts in NOD/SCID Mice Allows to Detect Differences among Vascular-Targeted Therapies

Informatic routines

Description and workflow of usage

Routines provided as supplementary material:

- S8_3DFilter.txt [the text of an ImageJ macro]
- S9_Vessel_Calibrometry.txt [the text of an ImageJ macro]
- S10_Spatial_Dispersion.java [the java code for an ImageJ plugin]
- S11_Spatial_Dispersion.txt [the text of an ImageJ macro]

3DFilter.txt

This ImageJ macro filters volumes in 3D using Analyze Particles and TransformJ. To operate it you need to download and install the TransformJ package (Erik Meijering: http://www.imagescience.org/meijering/software/transformj/). This macro filters an input binary stack and removes groups of connected voxels from particles whose Cartesian section is less than a surface threshold choosen by the operator. According to the modality of operation (OR/AND) the macro removes voxels only from sections filtered in every Cartesian plane (OR modality) or from sections filtered in at least one Cartesian plane (AND modality).

Usage

Download and install the full TransformJ package (Erik Meijering:

http://www.imagescience.org/meijering/software/transformj/). Then run this macro using the ImageJ "Run macro" command and selecting its file. At start the operator will have the possibility to choose the surface thresholds in the 3 Cartesian planes and the filter modality (OR/AND) The macro will ask for the folder grouping all the volumes to be filtered (one by one). Then, the macro will ask for a target folder where to save the filtered volumes.

Vessel_Calibrometry.txt

This ImageJ macro filters volumes in 3D using Analyze Particles and TransformJ. This macro identifies vessels of different caliber in square-based image stacks and map them to 6 new output stacks on the basis of a set of predefined thresholds. As a first step this macro filters an input stack removing all surfaces less than an increasing set of thresholds and saving a full stack for every filtering step. This passage is performed in 3D rotating the input volume by means of the TransformJ utility and using the standard ImageJ Analyze Particles command to perform filtration on each Cartesian plane. Filtered stacks are then tilted back to their original orientation and merged using the intersection operator AND.

Serial subtraction of increasingly filtered volumes provides a set of stacks mapping the location of vascular components with cross sections comprised between 2 consecutive filter thresholds. In this macro, thresholds are predefined in geometrical sequence starting with the value of 4 (values 4, 8, 16, 32, 64, 128, 256). As a function of the scale of the image, these thresholds may represent vessels with different calibers and functions.

<u>*Usage*</u>

As told precedently, download and install the full TransformJ package (Erik Meijering: http://www.imagescience.org/meijering/software/transformj/). Then run this macro using the ImageJ "Run macro" command and selecting its file. The macro will ask for a folder grouping all the volumes to filter (one at a time). Note that, for file managing reasons, the name of each

volume must contain a trait ("-") and the portion on the left of the trait must be univoque. Then, the macro will ask for a target folder where to save the filtered volumes.

The macro, which is rather slow, will prepare 2 subfolders for each filtered volume, named on the basis of the input volume. In the first subfolder, characterized by a "bp" tag, the macro will group the final, filtered and subtracted, stacks grouping vessels characterized by a close caliber (cross section). In the accompanying folder, the macro will save the intermediate volumes.

Spatial_Dispersion.java

Given a set of isotropic, equidimensional, binary stacks of squared images, this plugin calculates the number of cycles of rhombicubocthaedral dilation necessary to fill 90%, 95% and 99% of each volume on the basis of the distribution of the black pixels. For details on 3D rhombicubocthaedral dilation see Figure S5.

Usage

Open the file and compile it using the ImageJ "compile and run" command. Copy the saved class into the plugin folder of the ImageJ application and restart ImageJ. Operate the plugin by selecting it from the ImageJ Plugins menu.

The plugin asks for a source folder containing a set of subfolders (mandatory) containing image stacks (inverted LUT) with the same dimensions . Note that the plugin accepts also a single subfolder containing a single stack. The procedure is iterated over the nested folders for all the images present in every subfolder. The results are saved in a target location, choosed by the operator, as a number of .txt result files, named after the source subfolders. A progress bar provide indications on the time needed before work completion.

Results are expressed as raw values (Hv) which are normalized, subfolder by subfolder and according to the highest percent volume observed among all images of each subfolder, to

give nHv results. To normalize all subfolders in a single run, the image presenting the highest percent volume should be copied in all subfolders. For every stack in a subfolder the result file report:

- the percent volume of the stack
- the raw number of cycles needed to fill 90%, 95% and 99% of the volume
- the normalized values calculated using the highest percent volume (listed separately)

This plugin requires at least ImageJ 1.43 and has been tested on version 1.44o.

Spatial_Dispersion.txt

This ImageJ macro is functionally identical to the above plugin and provide identical results. It is operated using the ImageJ "Run macro" command and selecting its file. The macro is much slower than the plugin and has been used mainly to validate the results obtained by it.

Usage

Again the macro asks for an input folder containing a set of subfolders. Subsequently it asks for a folder where to save results. A progress bar provide indications on the time needed before work completion.
