# Supplementary figures and images for: D Quantification of Tumor Vasculature in Lymphoma Xenografts in NOD/SCID Mice Allows to Detect Differences among Vascular-Targeted Therapies
Source: PLoS One. 2013 Mar 26;8(3):e59691. doi: 10.1371/journal.pone.0059691 (PMC3608557; doi:10.1371/journal.pone.0059691)

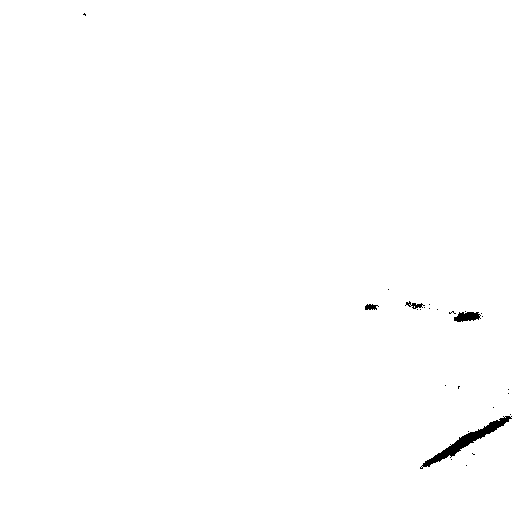

Supplement: Supporting Information S12 — A compressed file containing a folder with 2 image files referring to different processing steps for an acquired image from an untreated KMS-11 tumor sample. The folder, that can be treated as a test data set, contains: a) Image binadjG12S_Ctrl1-iso.tif which is the binary image resulting from Renyi Entropy thresholding of an isotropic, contrast-enhanced, 8-bit stack ready to be turned binary. b) Image FOR4baG12S_Ctrl1-iso.tif which is the binary image after removal of particles with Cartesian sections less than 1 µm2 (4 px). This last image is ready to be splitted into subimages, according to vessel cross-sections, using macro Vessel_Calibrometry.txt. (ZIP) [file pone.0059691.s012.zip › S12_ControlImage/binadjG12S_Ctrl1-iso.tif]

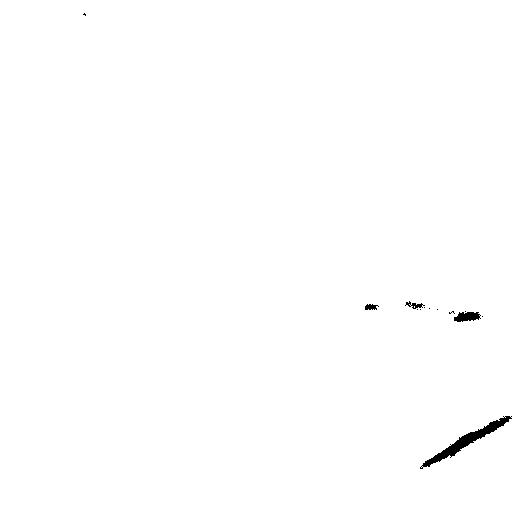

Supplement: Supporting Information S12 — A compressed file containing a folder with 2 image files referring to different processing steps for an acquired image from an untreated KMS-11 tumor sample. The folder, that can be treated as a test data set, contains: a) Image binadjG12S_Ctrl1-iso.tif which is the binary image resulting from Renyi Entropy thresholding of an isotropic, contrast-enhanced, 8-bit stack ready to be turned binary. b) Image FOR4baG12S_Ctrl1-iso.tif which is the binary image after removal of particles with Cartesian sections less than 1 µm2 (4 px). This last image is ready to be splitted into subimages, according to vessel cross-sections, using macro Vessel_Calibrometry.txt. (ZIP) [file pone.0059691.s012.zip › S12_ControlImage/FOR4binadjG12S_Ctrl1-iso.tif]
